# Pentamers Not Found in the Universal Proteome Can Enhance Antigen Specific Immune Responses and Adjuvant Vaccines

**DOI:** 10.1371/journal.pone.0043802

**Published:** 2012-08-24

**Authors:** Ami Patel, Jessica C. Dong, Brett Trost, Jason S. Richardson, Sarah Tohme, Shawn Babiuk, Anthony Kusalik, Sam K. P. Kung, Gary P. Kobinger

**Affiliations:** 1 Special Pathogens Program, National Microbiology Laboratory, Public Health Agency of Canada, Winnipeg, Canada; 2 Department of Medical Microbiology, University of Manitoba, Winnipeg, Canada; 3 Department of Immunology, University of Manitoba, Winnipeg, Canada; 4 Department of Computer Science, University of Saskatchewan, Saskatoon, Canada; 5 National Centre for Foreign Animal Disease, Canadian Food Inspection Agency, Winnipeg, Manitoba, Canada; University of Pittsburgh, United States of America

## Abstract

Certain short peptides do not occur in humans and are rare or non-existent in the universal proteome. Antigens that contain rare amino acid sequences are in general highly immunogenic and may activate different arms of the immune system. We first generated a list of rare, semi-common, and common 5-mer peptides using bioinformatics tools to analyze the UniProtKB database. Experimental observations indicated that rare and semi-common 5-mers generated stronger cellular responses in comparison with common-occurring sequences. We hypothesized that the biological process responsible for this enhanced immunogenicity could be used to positively modulate immune responses with potential application for vaccine development. Initially, twelve rare 5-mers, 9-mers, and 13-mers were incorporated in frame at the end of an H5N1 hemagglutinin (HA) antigen and expressed from a DNA vaccine. The presence of some 5-mer peptides induced improved immune responses. Adding one 5-mer peptide exogenously also offered improved clinical outcome and/or survival against a lethal H5N1 or H1N1 influenza virus challenge in BALB/c mice and ferrets, respectively. Interestingly, enhanced anti-HBsAg antibody production by up to 25-fold in combination with a commercial Hepatitis B vaccine (Engerix-B, GSK) was also observed in BALB/c mice. Mechanistically, NK cell activation and dependency was observed with enhancing peptides ex vivo and in NK-depleted mice. Overall, the data suggest that rare or non-existent oligopeptides can be developed as immunomodulators and supports the further evaluation of some 5-mer peptides as potential vaccine adjuvants.

## Introduction

The breadth and amplitude of an immune response can be related to how frequently a specific amino acid sequence is found in nature [1]. Antigens from infectious agents that are highly immunogenic are more likely to express peptide sequences that are less common in the human proteome [2]. In this way, exotic amino acid sequences that are rarely encountered can generate robust immune responses, allowing the host to mount strong defences against uncommon invaders [3]–[7].

Bioinformatics tools can be used to probe the frequency of different lengths of oligopeptides in the universal proteome database as represented by UniRef100 (http://www.uniprot.org). This analysis revealed that all possible 4 amino acid (aa) peptide combinations occur at least once in humans and all other organisms. Interestingly, contrary to statistical predictions, certain 5 aa and 6 aa peptide combinations are absent from all publicly available proteome sequences [8], [9]. Short 5–6 aa sequences have been shown to be important in the functional activity of enzymes, cell growth and hormone regulation, transcript expression, proteases, epitope binding, and immune activation [3], [8], [10], [11]. This suggests that short peptide sequences that are not found in humans, other mammals, or other organisms could have biological function; if incorporated, for example into existing vaccines or other therapies. Combining vaccine candidates with immunomodulatory peptides has previously been shown to enhance immunogenicity by facilitating immune cell interactions [3], [12]–[14].

The current study investigated the potential immunomodulatory activity of several short 5 aa peptides (also known as pentamers or 5-mers) that are not found in humans and are not found or are extremely rare in other organisms. Additional 9 aa (9-mer) and 13 aa (13-mer) peptides consisting of 5-mer repeats better fitting in the major histocompatibility class (MHC) class I and II binding grooves were also evaluated as candidates. Each peptide was initially incorporated onto the end of an H5N1 influenza hemagglutinin (HA) protein as a prototype antigen. These constructs were evaluated in parallel with a well-characterized H5N1-HA DNA vaccine in mice [15] for their ability to induce immune responses and protection against H5N1. The efficacy of the most promising 5-mer was evaluated as an exogenous (free) peptide combined in solution with H5N1 or H1N1 HA DNA vaccines in mice and ferrets. The 5-mer was also evaluated with a commercial Hepatitis B vaccine currently widely used in humans. Exploiting the concept of robust immune responses stimulated from rare exotic antigens, we describe here the generation, *in vivo* evaluation, and identification of a novel class of short peptides with immunomodulatory activity and potential adjuvant effects.

## Results

### In Silico Scanning of the Universal Proteome Database for Rare Short Peptides

The entire universal proteome was accessed through the UniRef100 database (http://www.uniprot.org) and a combination of UNIX/LINUX shell scripts and Perl programs was used to determine the frequency of all possible 5-mer peptide sequences in all natural kingdoms of life. 5-mer peptides were selected as the focus of the current study since the list of non-existent 6-mer peptides is considerably greater due to the increased number (20 times) of possible combinations of six amino acids. The total number of 5-mers versus peptide occurrence was then plotted in order to represent graphically the distribution of 5-mers in the universal proteome (Figure S1). Two hundred rare, 200 semi-common, and 200 common 5-mer peptides were randomly selected as an initial test to evaluate differences in immune modulation with respect to peptide frequency.

**Figure 1:**
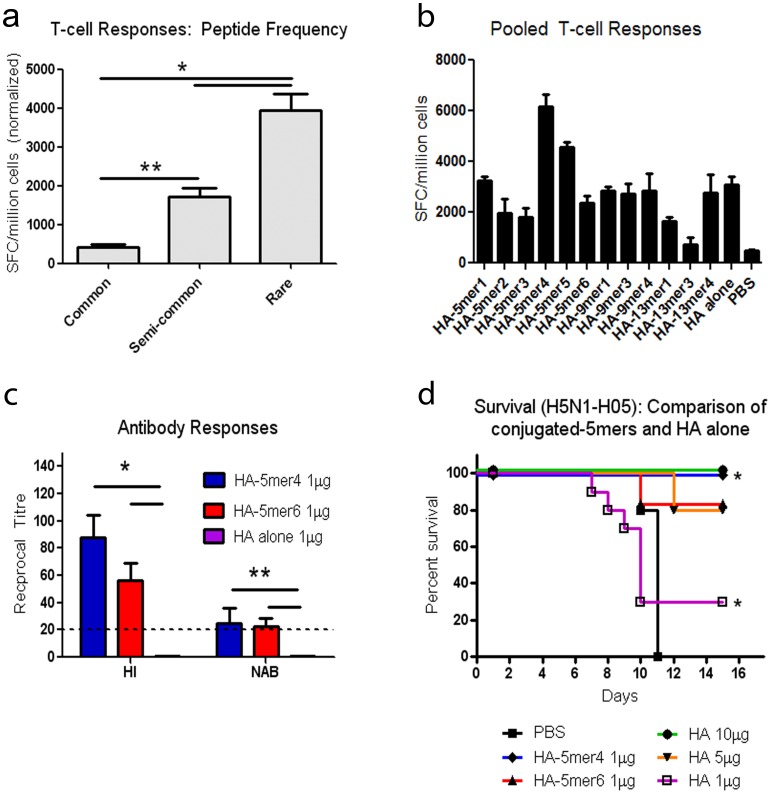
Immune responses and protection following conjugation of rare occurring peptides to the H5N1-HA antigen C-terminus. Individual 5-mer, 9-mer, or 13-mer peptides consisting of amino acids that occur rarely or never in sequence were added to the end of the H5N1-HA antigen by PCR. (a) Pooled T-cell immune responses generated by rare, semi-common, and common peptides. Groups of 3–4 BALB/c mice were immunized with pools of 10 peptides representing 200 rare, 200 semi-common, and 200 common peptides and cellular immune responses were detected 10 days post-vaccination by visualization of IFN-γ secretion by spot forming cells (SFC). The data, representing the average SFC in each category of peptide, is representative of 3 independent experiments and is normalized based on comparison with the HA DNA vaccine alone to distinguish changes with respect to peptide frequency. The average SFC baseline from unstimulated control splenocytes is (40±8). (b) T-cell responses following vaccination. Groups of 4 BALB/c mice were immunized with 50 µg of each HA-peptide construct and cellular immune responses were detected 10 days post-immunization. (c) Hemagglutination inhibition and neutralizing antibody responses at day 25 post-vaccination. Serum was individually evaluated from 8–10 BALB/c mice immunised with HA-5mer4 (1 µg), HA-5mer6 (1 µg), or HA alone (1 µg). HI antibody titres were detected using horse red blood cells. NAB titres were evaluated by monitoring MDCK cells for the presence of CPE. The dotted line represents the limit of detection of the HI and NAB assays. (*p<0.0001, **p<0.0001) (d) Survival against lethal H5N1-H05 challenge. Groups of 10 BALB/c mice were immunized with control PBS ▪, HA-5mer4 (1 µg) ♦, HA-5mer6 (1 µg) ▴, or H5N1-HA (1□, 5 ▾, or 10 µg •) and then challenged with 100LD_50_ of homologous H5N1-H05 virus. Animals were monitored over a period of 15 days. (*p<0.01) Error bars represent the standard error of the mean (SEM).

First, a DNA vaccine expressing the avian influenza H5N1 hemagglutinin gene (H5N1-HA) was selected to evaluate the effect of each category of 5-mer peptides on immune responses to a viral antigen. The ELISPOT IFN-γ assay was chosen as an efficient and rapid method to monitor modulation of the cellular response, representing one arm of the adaptive immune response. Rare, semi-common, or common 5-mers were first combined in pools of 10 peptides in order to facilitate initial screening using an *in trans* approach to detect potential immunogenic peptides Groups of 3–4 BALB/c mice were immunized with the H5N1-HA DNA vaccine construct (50 µg) combined with each peptide pool *in trans* as exogenous (free) peptide (50 µg total peptide). T-cell responses were assayed 10 days post-vaccination. Rare 5-mer peptides generated significantly higher total T-cell responses in comparison with semi-common and commonly occurring peptides (Figure 1a, * and ** p<0.0001).

To further explore this tendency, a list of 1705 5-mers not found in the universal proteome was generated and six peptides were selected at random within a range of hydrophobicity from 20–60% for evaluation of their ability to modulate immune responses (Table S1). A list of 9-mers and 13-mers, made from 5-mer subunits not found in the universal proteome, was also generated to address whether increasing the length of the peptides to better fit within the MHC class I or II binding groove would result in improved immunomodulation. Since this study was initiated, each of the selected 5-mers has been reported in at least one organism in the universal proteome according to a BLASTP search at NCBI (Table S1). The occurrences are usually within hypothetical proteins, typically with a single occurrence in each organism. None of the 5-mers have been described in the human proteome.

### Evaluation of Immune Responses Induced by Selected Rare Peptide Sequences Conjugated to an H5N1-HA DNA Vaccine in a Mouse Model of Influenza Infection

Initially, each 5-mer, 9mer, and 13-mer peptide sequence was cloned downstream of the hemagglutinin (HA) gene of H5N1 A/Hanoi/30408/2005 (H05), in frame at the C-terminus, generating a chimeric fusion protein for expression of the short peptides *in cis* with the antigen. The H5N1 model presented the additional advantage of offering the possibility to perform challenge studies and therefore evaluate the effect of these short peptides on protection against lethal infection *in vivo*. The *in cis* approach also ensured delivery of the short amino acid sequences together with the HA antigen *in situ* with an equimolar ratio of the HA antigen to short peptide. Western blot analysis was performed and showed that all constructs were expressing the H5N1-HA fusion proteins at comparable levels (Figure S2).

To evaluate the ability of the low frequency peptides to modulate immune responses, groups of 10 BALB/c mice were vaccinated with each H5N1-HA-peptide construct and screened for changes in T-cell activation and antibody responses. Mice were immunized with 50 µg of the HA-peptide constructs and T-cell responses were monitored by ELISPOT-IFN-γ assay. A significant increase in IFN-γ secreting cells was detected following immunization with HA-KWCEC (5mer4, 50 µg) compared with HA (50 µg) (Figure 1b; p<0.05). Responses detected with the other 5-mers, 9-mers, and 13-mers were not statistically different than with HA alone. Antibody responses against the H5N1-H05 virus were detected by hemagglutination inhibition (HI) and neutralizing antibody (NAB) assays. Significantly higher HI and NAB titres were detected following vaccination with HA-5mer4 (1 µg) in comparison with HA alone, which did not have detectable antibody responses at a 1 µg dose (Figure 1c, p<0.001). Vaccination with HA-5mer6 (1 µg) also offered significantly higher antibody titres despite stimulating a comparable T-cell response to HA alone.

Finally, the HA-5mer4 fusion was evaluated *in vivo* as a potential vaccine candidate against lethal homologous H5N1-H05 challenge in groups of 10 BALB/c mice. A single 1 µg dose of HA-5mer4 afforded 100% protection, whereas 30% protection was observed with 1 µg of the HA DNA vaccine (Figure 1 d, p<0.01). HA-5mer6 (1 µg) was also evaluated since it generated strong antibody titres post-vaccination and offered 80% protection in the same conditions. Overall, the 5mer4 short peptide resulted in the highest increase in immune responses against the HA antigen and best protection against lethal challenge with H5N1 in mice. 5mer4 was therefore selected for evaluation in further experiments.

### Protection in Mice and Ferrets during Lethal Influenza Virus Challenge with the HA DNA Vaccine Mixed with Exogenous 5mer4 Peptide

**Figure 2:**
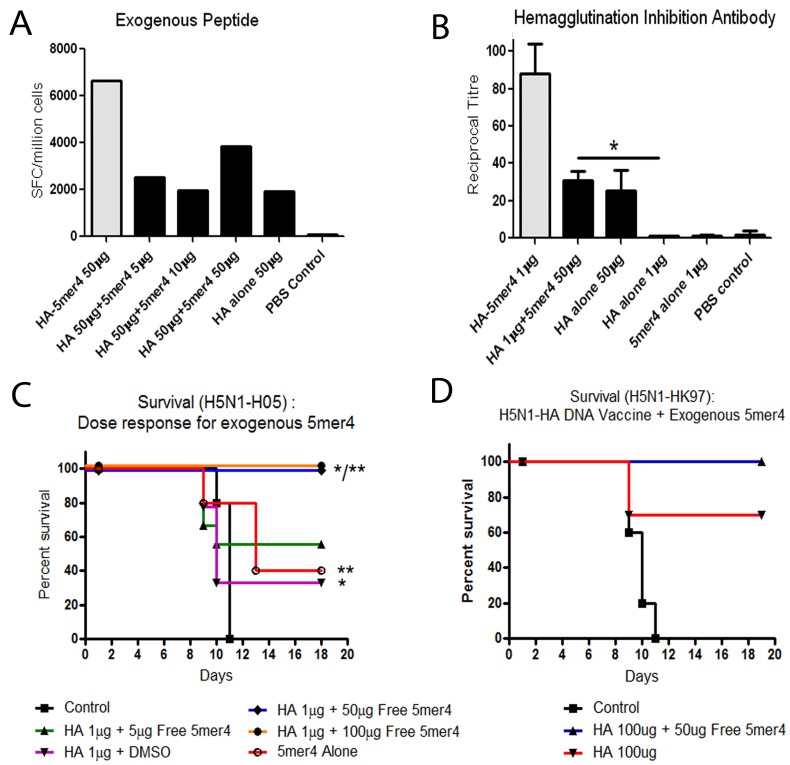
Immune responses and protection following addition of exogenous 5mer4 to the H5N1-HA DNA vaccine. Exogenous 5mer4 was added to the H5N1-HA DNA vaccine and administered to groups of BALB/c mice. (a) T-cell responses. Groups of 4 BALB/c mice were immunized with H5N1-HA DNA vaccine (50 µg) and increasing concentrations of 5mer4 adjuvant (5, 10, or 50 µg). Cellular immune responses were screened 10 days post-immunization through detection of IFN-γ secretion. Responses from the HA-5mer4 conjugate vaccine (Figure 1b) are presented in grey for comparison. (b) Hemagglutination inhibition antibody responses. Serum was individually evaluated from 8–10 BALB/c mice immunized with 5mer4 (50 µg), H5N1-HA (1 µg) + 5mer4 (50 µg), H5N1-HA (1 µg), H5N1-HA (50 µg), or PBS. HI antibody responses were detected using horse red blood cells. (*p = 0.004) The HI antibody titre from the HA-5mer4 conjugate vaccine (Figure 1c) is presented in grey for comparison. (c) Survival against lethal homologous challenge. Groups of 10 BALB/c mice were vaccinated with H5N1-HA (1 µg) + 5mer4 (5 ▴, 50 ♦, or 100 µg •), 5mer4 (50 µg ○), H5N1-HA (1 µg ▾) + DMSO, or a PBS control ▪. Animals were challenged with 100LD_50_ of lethal homologous H5N1-H05 virus and monitored for 18 days. (*p = 0.006, **p<0.01) (d) Survival against lethal heterologous challenge. Groups of 10 BALB/c mice were vaccinated with H5N1-HA (100 µg) + 5mer4 (50 µg) ▴, H5N1-HA (100 µg) ▾, or a PBS control ▪. Animals were infected with 100LD_50_ of heterologous H5N1-HK97 virus. Error bars represent ± SEM.

The 5mer4 peptide was added *in trans* in solution with the DNA vaccine in order to evaluate whether or not this alternative approach could offer comparable levels of protection to *in cis* delivery. Different doses of exogenous peptide (5, 10, and 50 µg) were mixed in solution with the H5N1-HA DNA vaccine and compared in parallel with H5-HA DNA conjugated vaccine (50 µg), HA-5mer4 DNA alone (50 ug), or unimmunized control animals (Figure 2a). T-cell responses following vaccination with exogenous 5mer4 peptide (50 µg) + H5N1-HA (50 µg) were increased compared to the HA DNA vaccine (50 µg). HI responses were also increased, between delivery of 5mer4 *in trans* and HA alone (Figure 2b, p = 0.004). HA-5mer4 conjugate vaccine data from Figure 1 is presented in grey for comparison.

Free 5-mer4 (50 µg) combined with the H5N1-HA DNA vaccine (1 µg) offered comparable protection against lethal homologous H5N1-H05 challenge to the HA-5mer4 fusion (1 µg) (Figure 2c). Unexpectedly, administration of the exogenous 5mer4 peptide with no vaccine afforded 40% protection, suggesting that despite the absence of a detectable HA-specific response, non-specific immune stimulation can prepare treated animals to better defend themselves against H5N1 infection. Both humoral and cellular immune responses were undetectable following administration of 5mer4 alone suggesting that it does not mimic HA-specific immune responses at a biologically significant level. Complete protection (100%) against lethal heterologous H5N1-HK97 challenge was also observed when 5mer4 (100 µg) was combined with the H5N1-HA DNA vaccine (100 µg) compared with a similar dose of HA alone (70%) (Figure 2d, p = 0.09).

**Figure 3:**
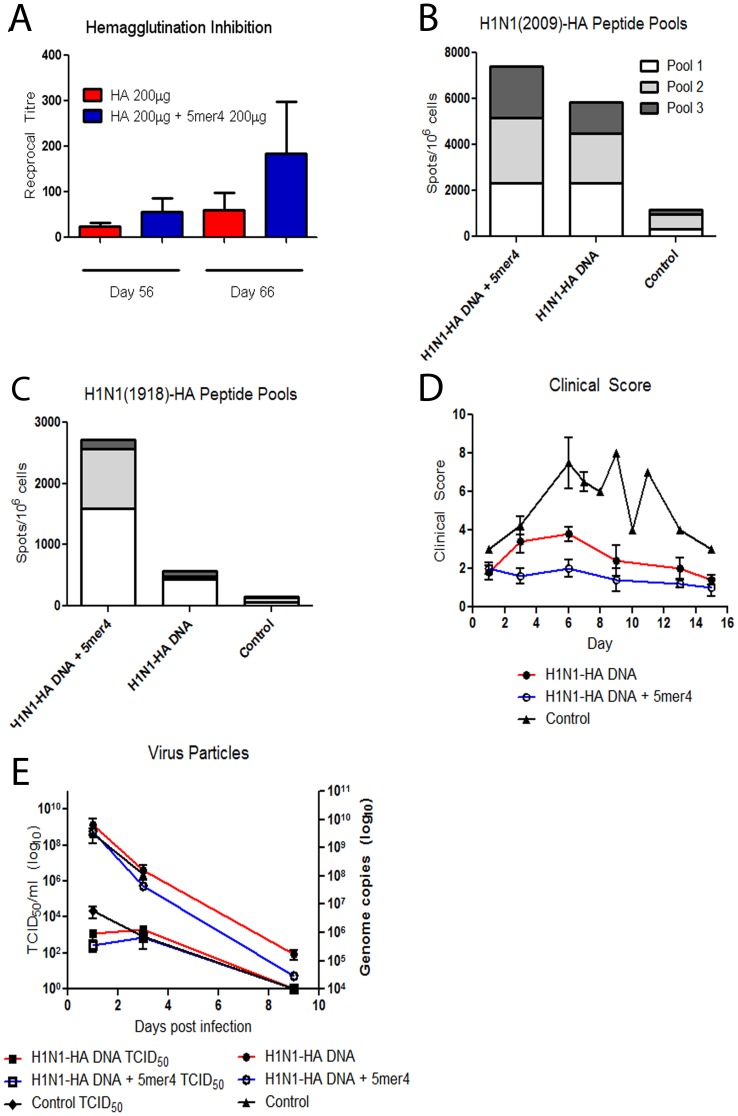
Evaluation of H1N1-HA DNA vaccine combined with exogenous 5mer4 adjuvant in ferrets. Ferrets were immunized with 200 µg of the H1N1-HA DNA vaccine with or without the 5mer4 (200 µg) adjuvant. (a) Hemagglutination inhibition antibody responses. Ferrets received immunizations with the H1N1-HA DNA vaccine, administered 3 times at one month intervals. Serum was collected after each vaccination and prior to challenge. HI antibody responses were evaluated at days 55 and day 66 post-immunization and detected using turkey red blood cells. (b) HA-specific T-cell responses. PBMCs were collected by ficoll gradient from all ferrets following challenge with the H1N1-2009 virus. T-cell responses were evaluated following re-stimulation with 3 peptide pools representing the H1N1-2009 HA protein. (c) HA-specific T-cell responses following re-stimulation with 3 peptide pools representing the H1N1-1918 HA protein. (d) Clinical observations of ferrets following H1N1-2009 challenge. Ferrets were scored on a scale of 1–10, where 0 = no signs of disease, 10 = most severe. (E) Infectious and total virus particles. Ferret nasal washes were collected at days 1, 3, and 9 following infection. Left axis: Infectious virus titre from the nasal washes of H1N1-HA + 5mer4 (□), H1N1-HA (▪), and control (♦) ferrets was detected by TCID_50_ assay. Right axis: Following RNA extraction, nasal wash samples were probed with H1HA specific primers using quantitative real-time RT-PCR. H1N1-HA + 5mer4 (○), H1N1-HA (•), and control (▴) ferrets. Error bars represent the SEM.

As well, exogenous peptide was added to an H1N1-2009 HA DNA vaccine and efficacy was evaluated in ferrets to test the efficacy of 5mer4 in a different animal species relevant to influenza infection. Ferrets display similar clinical symptoms as humans, including disease progression, viral pathogenesis, and immune responses [16] (reviewed in [17] and [18]). It is, therefore, a good pre-clinical model to further assess the efficacy of combining 5mer4 with an HA vaccine and a system to measure changes in humoral and cellular immune responses. Each ferret received three immunizations at monthly intervals with H1N1-2009 HA DNA (200 µg) with or without free 5mer4 peptide (200 µg). The ferrets were then challenged intranasally with 10^6^ pfu per animal of homologous A/InDRE4487/2009 (H1N1) virus. On average, a modest increase in HI antibody titres was detected in the serum of animals vaccinated with H1N1-HA + 5mer4 after the last vaccination time point (day 55) and prior to challenge (day 66) (Figure 3a). T-cell responses were also monitored by ELISPOT-IFNγ assay from peripheral blood mononuclear cells (PBMCs) following vaccination [19]. Ferrets receiving H1N1-HA + 5mer4 had slightly higher HA-specific T-cell responses against H1N1 (2009), and interestingly a more robust increase against the heterologous H1N1 (1918)-HA pools (Figure 3b, c) compared to ferrets vaccinated with H1N1-HA alone.

The A/InDRE4487/2009 (H1N1) virus causes morbidity and an average of 48% mortality in ferrets [20]. Although all vaccinated animals survived the challenge, significant differences in clinical symptoms of disease at early time points were noted between the animals immunized with H1N1-HA + 5mer4 and those receiving H1N1-HA alone (Figure 3d). Infectious virus titres in nasal washes collected at 1, 3, and 9 days post-infection were detected by a TCID_50_ assay. Ferrets immunized with H1N1-HA + 5mer4 had an average 4.7-fold decrease in infectious virus titre on day 1 post-infection in comparison with H1N1-HA DNA alone (Figure 3e, left axis, p = 0.24)). Quantitative realtime RT-PCR was also used to detect the total H1N1-2009 genome copies in the nasal washes of vaccinated and control ferrets. At days 3 and 9 post-infection, an average of 3.6 times (p = 0.21) and 5.8 times (p = 0.07) lower copy numbers were detected, respectively, in the nasal washes of the H1N1-HA 5mer4 vaccinated group versus H1N1-HA (Figure 3e, right axis).

### Combination of the 5mer4 Pentapeptide with a Commercial Hepatitis B Vaccine

**Figure 4:**
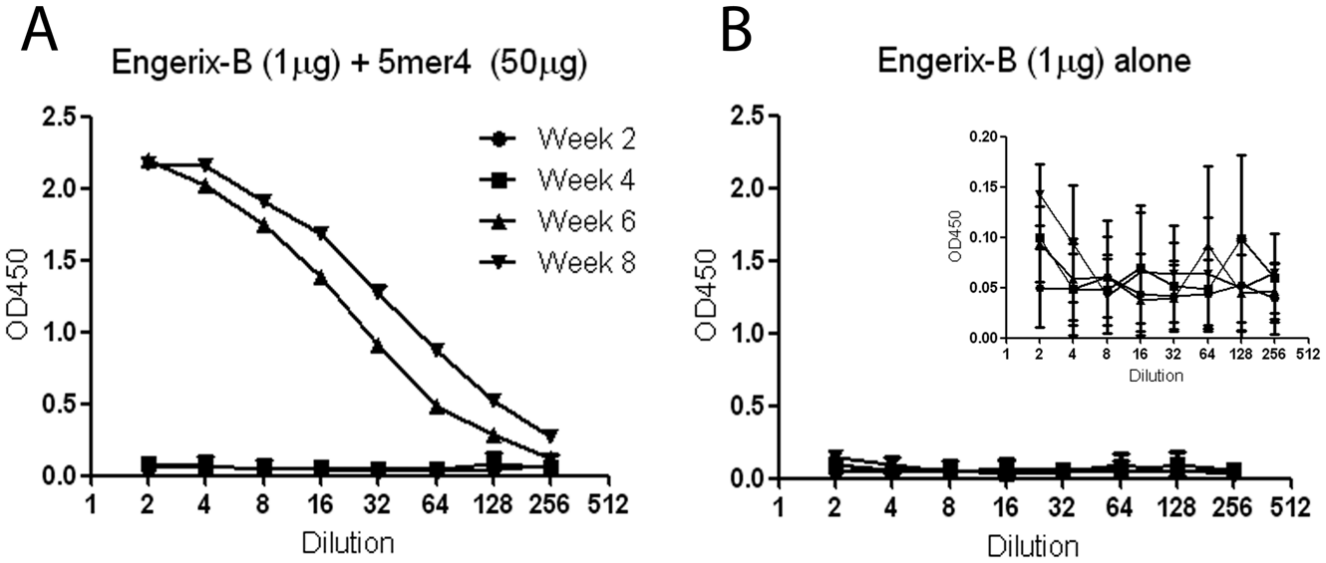
Efficacy of 5mer4 in combination with HBsAg. Exogenous 5mer4 (50 µg) was combined with a commercial Hepatitis B vaccine (Engerix-B, 1 µg). Control mice received the commercial vaccines without 5mer4. Serum was collected from vaccinated animals at 2 •, 4 ▪, 6 ▴, and 8 ▾ weeks post-immunization and evaluated in two-fold dilutions for detection by ELISA. Antibody levels were detected at OD450 nm. (a) Anti-HBsAg antibodies following vaccination with Engerix-B + 5mer4. (b) Anti-HBsAg antibodies following vaccination with Engerix-B alone. Inset displays the data within a more narrow y-axis range. The data represents the arithmetic mean titre ± SEM.

To evaluate the efficacy of 5mer4 in combination with another antigen, the 5mer4 peptide was combined with a commercial Hepatitis B vaccine (Engerix-B, GSK), which is adsorbed to aluminum hydroxide (alum). A group of 10 BALB/c mice was immunized with the Engerix-B vaccine (1 µg hepatitis B virus surface antigen (HBsAg) dose) combined with 5mer4 (50 µg dose). Serum was collected at 2, 4, 6, and 8 weeks post-immunization and screened for total anti-HBsAg antibodies by ELISA. Antibody titres were higher by up to 24-fold and 15-fold after 6 and 8 weeks, respectively, at the lowest dilution for the 5mer4 + HBsAg combination (Figure 4a) compared to HBsAg alone (Figure 4b) (p<0.0001).

**Figure 5:**
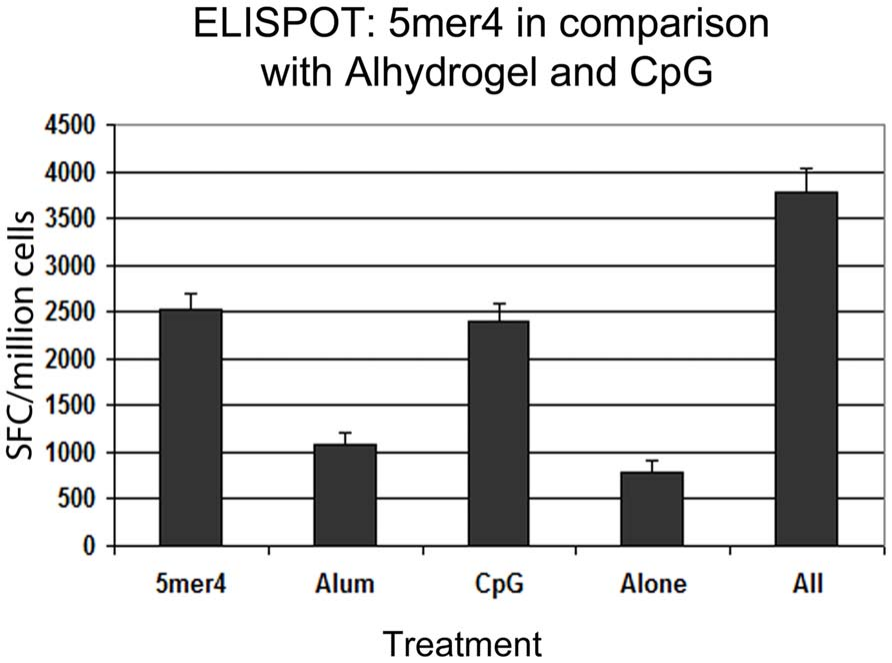
Comparison of 5mer4 with other commercial adjuvants. Groups of 3–4 BALB/c mice were immunized with H5N1-HA (50 µg) alone, H5N1-HA (50 µg) + 5mer4 (50 µg), H5N1-HA (50 µg) + CpG (10 µg), H5N1-HA (50 µg) adsorbed to Alhydrogel (450 µg), or “All” (H5N1-HA + 5mer4 (50 µg) + CpG (10 µg) + adsorbed to Alhydrogel (450 µg). Cellular immune responses were monitored 10 days post-immunization. Splenocytes were re-stimulated with H5N1-HA peptides pools and detected for IFN-γ secretion. The bars represent total pooled T-cell responses. Error bars represent ± SEM.

Together, these results suggest that 5mer4 can positively modulate immune responses and improve the immunogenicity of the Hepatitis B vaccine. Therefore, 5mer4 was compared with other known adjuvants to assess its efficacy and to evaluate whether combining it with previously described adjuvants could be additive or synergic. The efficacy of 5mer4 was compared in parallel with Class C CpG ODN2395 (Invivogen) and alum (Alhydrogel). CpG ODN is a TLR9 ligand; however, different pathways and subsequent activation of immune cells may be stimulated depending on the specific oligodinucleotide sequence 21–23. Mice were immunized with 50 µg H5N1-HA DNA vaccine combined with 10 µg of CpG ODN or adsorbed to 450 µg of alum. T-cell responses were evaluated following re-stimulation of splenocytes with H5N1-HA peptide pools and monitoring of IFN-γ production by ELISPOT. 5mer4 offered comparable levels of T-cell responses to CpG ODN (Figure 5, p = 0.63). Alum did not significantly improve T-cell response, a result consistent with previous studies evaluating the effect of alum on DNA-based vaccines [24]. Interestingly, the combination of H5N1-HA and 5mer4 (50 µg) + CpG (10 µg) + alum (450 µg) resulted in an additional increase in IFN-γ positive cells (p = 0.052 in comparison with 5mer4).

### The Immunomodulation by 5mer4 is NK Cell-dependent

Partial survival against a uniformly lethal challenge with H5N1 influenza virus was observed when 5mer4 was administered alone, suggesting antigen-non-specific activation of the immune system leading to an enhanced response against the H5N1 virus. We examined whether non-specific molecular pattern recognition receptors and/or innate cells such as macrophages, DC, or NK cells were involved in the immune responses induced by the 5mer4 peptides *in vivo*.

Individual toll-like receptors (TLR) or nod-like receptors (NLR) were expressed *in vitro* in an HEK 293 reporter system to detect association with 5mer4 peptide induced pathways (Invivogen). Different concentrations of peptide (0.05, 0.5, 5, or 50 µg/ml) were evaluated, however, no significant activation was detected using NF-κB as a readout (data not shown).

Primary cultures of bone marrow-derived dendritic cells, macrophages, or splenic NK cells were evaluated for changes in activation marker expression in the presence or absence of 5mer4 *in vitro*. Cells were incubated with 5mer4 alone or in combination with the H5N1-HA DNA vaccine, as well as with the peptide diluent DMSO or unrelated peptide 5mer2 as controls. No up-regulation of activation markers was observed following incubation with bone-marrow derived immature (CD11c^+^, CD1a^+^, CD80^−^, CD86^−^) or mature dendritic cells (CD11c^+^, CD80^+^, CD86^+^) (data not shown). Bone marrow-derived macrophages (CD11b^+^, F4/80^+^, CD11c^−^) also did not display differential phenotypic changes characteristic of activation in the presence or absence of 5mer4 with no increase in CD80/CD86 (data not shown).

**Figure 6:**
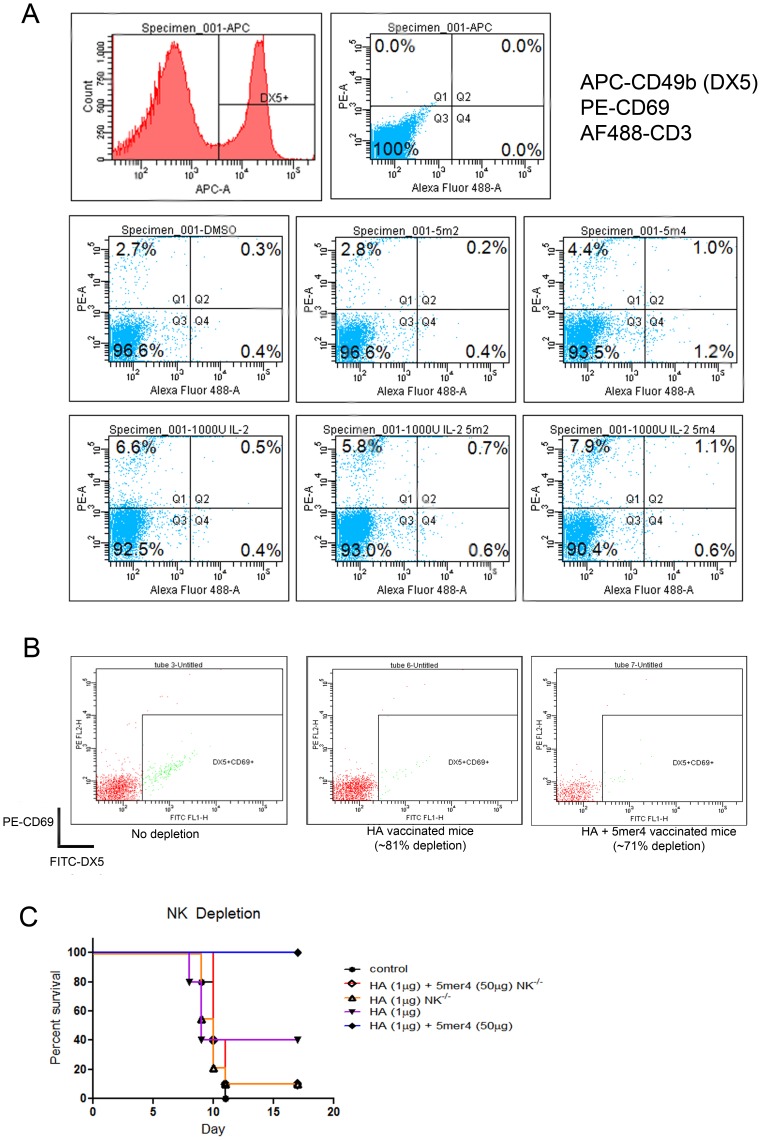
Evaluation of NK cell responses in response to 5mer4. (a) NK cells were isolated from the spleen of BALB/c mice using negative selection. Cells were incubated overnight in the presence of 100 U/ml recombinant IL-2, with 2.5 µg/ml of 5mer4, 5mer2 control, DMSO control. Additional groups of cells were incubated with 1000 U/ml IL2 and 1000 U/ml IL2 combined with 5mer4 (2.5 µg/ml). NK cell activation was monitored for PE-CD69 upregulation on APC-DX5^+^ cells by flow cytometry. All cells were CD3^−^. The data is representative of three independent experiments performed on different days. (b) NK depletion in BALB/c mice. Mouse NK cells were depleted by administration of anti-asialo antibody at day -2 and day -1 before vaccination. Whole blood samples were collected from the saphenous vein (50 µl) from NK-depleted and undepleted control animals. All samples were first treated with ACK lysis buffer to remove red blood cells and then stained with PE-CD69 and FITC-DX5 to detect the percentage of NK cells remaining following depletion. (c) Survival of vaccine groups following NK cell depletion. At day 0, NK-depleted BALB/c mice were immunized with H5N1-HA (1 µg) or H5N1-HA (1 µg) + 5mer4 (50 µg), or PBS control. Three groups of mice that were not depleted were immunized with H5N1-HA (1 µg), H5N1-HA (50 ug) or H5N1-HA + 5mer4 (50 µg) as controls. Animals were challenged with 100LD_50_ of homologous H5N1-H05 virus and survival was monitored over 17 days. Groups of 8–10 BALB/c mice were evaluated.

In contrast, the addition of the 5mer4 peptide (2.5 µg/ml) to purified CD3^−^NK cells significantly increased the activation marker CD69 when compared to control DMSO or with the unrelated 5mer2 peptide, which had not demonstrated immune modulation activity (Figures 1 and 6). Additionally, pre-activated NK cells (using 1000 U/ml IL2) exhibited enhanced activation and upregulation of surface markers as observed through expression of CD69 and DX5 (CD49b) markers.

To further assess the significance of the interaction between NK cells and peptide 5mer4 *in vivo*, NK cells were depleted from BALB/c mice prior to vaccination with H5N1-HA with or without the 5mer4 peptide (Figure 6b). The enhanced vaccine efficacy phenotype was not observed in 5mer4 treated mice following NK cell depletion (Figure 6c).

## Discussion

This study followed the hypothesis that rare or non-existent 5-mer amino acid sequences could act as immune modulators positively contributing to antigen-specific immune activation and adjuvant vaccines. Initial evaluation of common, semi-common and rare peptide pools further supported the concept of enhanced immune modulation with rare or semi-common versus common peptide sequences of identical length. Indeed, evaluation of pools of 200 rare, semi-rare or commonly found 5-mer sequences provided further experimental evidence linking rarity of occurrence and immune activation. The present study also shows that selected rare 5-mer sequences could positively modulate the immune response. Interestingly, administration to mice of 5mer4 alone resulted in partial survival against a uniformly lethal challenge with H5N1 influenza virus, suggesting antigen-non-specific activation of the immune system leading to an enhanced response against the H5N1 virus. Remarkably, of the six 5-mers sequences selected randomly from a set of 1705, two resulted in an enhanced antigen-specific immune response when administered in combination with a DNA-based vaccine expressing HA.

This study demonstrated that incorporation of 5-mer peptides onto the C-terminus end of the H5N1-HA protein does not affect protein expression. It is possible that translation of the conjugated 5-mer (*in cis)* may modulate antigen-specific (HA) immune priming more efficiently being in a 1 to 1 molar ratio from intracellular expression. One group of scientists also introduced non-occurring peptide sequences into native proteins and observed little impact on overall protein expression, folding, and stability [25]. Another report suggested that the heat of formation of rare and non-occurring 5-mer peptides is not significantly different than more commonly occurring sequences [26]. The rate of energy expenditure appears to be restricted to a smaller range, indicating that natural peptide synthesis and assembly are not limiting factors and that there must be other biological reason that explain why certain 5-mer peptides do not occur in living organisms.

The 5mer4 peptide also enhanced immune responses when combined with a commercial hepatitis B vaccine. Typically, the hepatitis B vaccine is administered in multiple boosts in order to ensure optimal seroconversion. Previous studies demonstrated the efficacy of combining CpG ODN with a conventional hepatitis B vaccine, avoiding the need for a boost in order to achieve detectable anti-HBS antibody titres [27]. Consistent with their study, anti-HBS antibodies were barely detectable following a single dose of the hepatitis B vaccine in mice without the peptide adjuvant. In this respect, it also seems unlikely that 5mer4 acts as a potential mimotope of H5N1-HA and cannot explain the range of enhanced immune responses following combination of 5mer4 with other vaccines such as HepB or other influenza subtypes (H5N1 and H1N1). One attractive feature of this strategy is the cost-benefit ratio for addition of a 5-mer peptide to a vaccine beside the significant dose sparing effect. On a large scale, the cost of a short peptide added to an existing commercial vaccine is less than one cent per vaccine dose (even taking into account the larger dose required for human use). This consideration could have important implications for broad vaccination campaigns, especially in developing countries where several vaccines are needed but resources are insufficient.

Although no visible signs of local inflammation or other side-effects were observed in mice or ferrets following administration of any of the 5-mer peptides, future work will need to formally evaluate toxicity and the exact range of antigens that will benefit from addition of short peptides like 5mer4 or other pentamers. Adjuvants facilitate interactions between innate immune cells, improving priming of adaptive humoral and cellular responses against an antigen. Adjuvant efficacy varies among antigens and the exact mechanism for several commercial adjuvant systems is still unknown. In this study, 5mer4 did not activate any cellular pattern recognition receptors that recognize generic motifs such as TLRs or NLRs, a finding also previously reported showing TLR-independent activity with other adjuvants [28]–[30]. Activation in mice was detected only from NK cells. Interestingly, recent studies have expanded the role of NK cells from their traditional importance as part of the innate immune response to include their influence on adaptive immune responses and the establishment of long-term memory [31] (reviewed in [32], [33]). The present data suggest that 5mer4 can activate NK cells resulting in an enhance adaptive immune responses as observed by increased T and B-cells responses and survival following challenge 28 days post-treatment. It will be necessary in future experiments to differentiate between the peptide- and antigen-specific immune responses and the roles that each play in the development of the adaptive immune response. Further evaluation of NK cell cytokines, pathways, and interactions with other innate and adaptive immune cells may provide a better overall picture for the mechanism of action of 5mer4 and other short immunomodulatory 5-mers.

At the start of this study, 1705 non-existing 5-mer peptides were identified from the universal proteome; however, this number decreased to 417 just recently [26]. All six of the 5-mer peptides initially selected for this study are now encoded in the proteome of one or more organisms (Table S1). None are found in the human proteome and peptide 5mer2 occurs only once in a mammal (platypus). It is important to note that the majority of the peptides are present in hypothetical proteins with no known function. Future investigations will need to clarify whether these amino acid sequences are effectively part of synthesized proteins.

The current study supports the concept that certain rare and non-existent short peptides can offer an alternative way to modulate immune responses Application of immune enhancing 5-mers as vaccine adjuvants may be particularly relevant for achieving better seroconversion and protection in young children and aging populations. Reduced antigen-specific cellular immune responses have been reported in elderly patients [34] and it is necessary to improve initial immune priming and the duration of antibody and memory T-cell responses post-immunization. Although studies suggest that total NK cell population increases in the elderly, it is reported that there is also an associated decrease in overall NK cell biochemical function and cytotoxicity [35]–[37] in addition to reduced long-term memory to antigen [38], [39]. Therefore, this could be one promising application of 5mer4 or other rare peptides to enhance innate immune responses. Alternatively, during the course of this study, we also identified several 5-mer peptides that reduced cellular immune responses (Figure 1b and data not shown). The exact biological consequence of rare 5-mer expression is not known but it is possible that the immune system has developed mechanisms of tolerance in the event of possible translation. However, it may be possible to incorporate these rare 5-mer peptides for treatment of conditions that require induction of tolerance or immune suppression (such as autoimmune disorders). Additionally, the human genome contains considerably large non-coding protein region of unknown function [40]. Many rare peptides may be encoded in the non-translated regions of these mRNA transcripts [41] or on the non-coding DNA strand [42]. Overall, the evaluation of 5-mer peptides that occur rarely or never in the universal proteome offers strong prospects for the development of novel immunomodulators and adjuvants against infectious diseases as well as for improving understanding of immune recognition.

## Materials and Methods

### Ethics Statement

All animal use, procedures, and scoring sheets were first approved by the Animal Care Committee at the Canadian Science Centre for Human and Animal Health, following the guidelines set by the Canadian Council on Animal Care.

### Cell Lines and Viruses

Human embryonic kidney (HEK) 293T cells were maintained on Dulbecco’s modified Eagles medium, containing 10% fetal bovine serum (FBS, Wisent) and 1% penicillin/streptomycin (pen/strep, Gibco). Madin-Darby canine kidney (MDCK) cells were maintained on minimum essential medium (MEM) supplemented with 10% FBS and 1% pen/strep.

The influenza A viruses A/Hanoi/30408/2005 (H5N1-H05), A/Hong Kong/483/1997 (H5N1-HK97), and A/Mexico/InDRE4487/2009 (H1N1-2009) were each amplified on MDCK cells maintained in MEM with 0.1% bovine serum albumin (BSA, Gibco) and 1% pen/strep in the presence of 1 ug/ml L-(tosylamido-2-phenyl) ethyl chloromethyl ketone-treated trypsin (TPCK-trypsin, Sigma). All virus titres were quantified by standard plaque assay on MDCK cells [15].

### In Silico Screening of Low Frequency Peptides

All publically available proteomes were accessed through the UniRef100 version of the UniProt database (http://www.uniprot.org) [43]. UniRef100 was chosen since it has duplicates and fragments eliminated, reducing the number of sequences that must be processed. Using a combination of UNIX/LINUX shell scripts and Perl programs, all sequences were scanned *in silico* to determine the frequency of each of the 20^5^ possible combinations of 5 aa. A list of 1705 rarely occurring or non-existent 5-mers was compiled and selected for further evaluation. Initially, several 5-mers were selected based on neutrality and mild hydrophobicity. A list of 9-mer and 13-mer candidates was also generated through concatenation of different combinations of overlapping non-occurring 5-mer subunits using a program written in the Prolog programming language.

Protein sequences are continuously being added to protein databases such as UniProt. To search for recent additions to the universal proteome that contain the selected 5-mers, it was convenient to use the BLASTP query tool. Unfortunately, the BLASTP tool at UniProt (http://www.uniprot.org/blast) was unable to search using 5-mers as queries. Hence, the BLASTP tool at NCBI (http://blast.ncbi.nlm.nih.gov/Blast.cgi) was employed. For the latter, the “nr” database for all organisms was used, and default parameters used.

### Peptides

All peptides and libraries for the experiments were obtained from commercial sources at >95% purity (Mimitopes, Australia and Genscript, New Jersey). Lyophilized peptides were resuspended in dimethyl sulfoxide (DMSO) at a final concentration of 100 µg/µl.

### Vaccines

A DNA vaccine expressing a codon-optimized H5N1-H05 hemagglutinin antigen (H5N1-HA) was generated from the A/Hanoi/30408/2005 H5N1 virus. The H5N1-HA is expressed under the control of a chicken beta-actin promoter and this DNA vaccine candidate has been previously shown to offer protection in mice against homologous and heterologous influenza virus challenges [15]. Individual 5-mer, 9-mer, or 13-mer peptides were added to the C-terminal end of the H5N1-HA gene by polymerase chain reaction (PCR) to prevent interference with the N-terminal signal peptide. Expression of HA with the added peptide sequences was confirmed by western blot using a polyclonal mouse anti-HA antibody and goat anti-mouse secondary antibody conjugated to horseradish peroxidise (HRP). BALB/c mice were immunized with 1 µg of H5N1-HA DNA for all challenge studies and 50 µg of H5N1-HA DNA for detection in T-cell assays. To evaluate the efficacy of exogenous, free peptide, 5, 10 or 50 µg of each 5-mer peptide was combined with each DNA vaccine in phosphate buffered saline (PBS). Mice were immunized with a single immunization of each vaccine (100 µl total volume, 50 µl in each hind limb.

A codon-optimized H1N1-2009 HA (H1N1-HA) vaccine was also constructed using the same DNA vector backbone for evaluation in a ferret model of influenza infection. Ferrets received an intramuscular dose of 200 µg of H1N1-2009 HA DNA vaccine with or without 200 µg of free peptide at three monthly intervals. All vaccines were administered diluted in PBS.

### Animal Models

Survival and immune responses were monitored in groups of BALB/c mice (6–8 weeks old, Charles River Canada). Groups of 8–10 mice were challenged with a 100LD_50_ dose of homologous H5N1-H05 or heterologous H5N1-HK97 viruses 28 days following vaccination. Each virus was diluted in virus diluent (MEM, 0.1% BSA, no antibiotics) and 50 µl was administered to each animal through the intranasal route. Animals were monitored for signs of disease and scored according to an approved endpoint chart over a period of 18–20 days. Ferrets (16–20 weeks) were immunized with the H1N1-HA DNA vaccine with or without peptide and challenged with 10^5^ 50% tissue culture infectious doses (TCID_50_) of H1N1-2009 virus by intranasal inoculation. Body temperature, weight changes, and signs of disease were monitored over a period of 14 days.

### T-cell Responses

An enzyme-linked immunosorbent spot (ELISPOT) assay was performed to detect T-cell responses following immunization. PVDF ELISPOT plates were coated with purified anti-mouse IFNγ (ELISPOT Mouse Set, BD Biosciences) overnight at 4°C. Groups of 3–4 BALB/c mice were immunized with the H5N1-HA DNA vaccines with and without peptide and spleens were harvested 10 days following vaccination. Splenocytes were then isolated by crushing spleens against a fine metal mesh in Leibovitz’s medium (L-15, Gibco) and filtered through a 40 µM cell strainer (BD Biosciences). Cells were then counted and resuspended in RPMI 1640 medium (supplemented with 10% FBS, L-glutamine, HEPES, non-essential amino acids, sodium pyruvate, pen/strep, and 5×10^−3^ molar β-mercaptoethanol) and then plated at 5×10^5^ cells/well. The harvested splenocytes were re-stimulated with 27 pools of overlapping peptides spanning the entire H5N1-H05 HA protein (final concentration of 2.5 µg/ml of each peptide). The H5N1-H05 HA peptide library consists of 112 overlapping peptides, 15aa in length with a 10aa overlap (Mimitopes, Australia). The library was then divided into the 27 peptide pools using a matrix format in order to efficiently screen immune responses [15]. The ELISPOT plates were then incubated overnight at 37°C with 5% CO_2_. T-cell responses were detected the following day by the presence of IFN-γ secretion on the PVDF membrane using a biotinylated anti-mouse IFN-γ antibody, followed by probing with streptavidin conjugated to HRP. IFN-γ secretion was detected using the AEC substrate kit (BD Biosciences) and read using an ELISPOT plate reader (AID ELISPOT reader). T-cell assays were performed at least in triplicate, except for initial screening where they were performed only once.

### Hemagglutination Inhibition (HI) Antibody Assay

Serum was collected from immunized and control animals at 25 days post-vaccination and treated with receptor-destroying enzyme (RDE, Accurate Chemical) at a 1∶3 ratio at 37°C for 18–20 hours. All samples were then incubated for 1 hour at 56°C to inactivate complement. Serum samples were diluted two-fold down a 96-well V-bottom microtitre plate, starting with a 1/10 dilution in PBS. Four hemagglutinating doses of H5N1-H05, H5N1-HK97, or H1N1-2009 were then incubated for 1 hour with the serum at room temperature. The serum-virus mixture was incubated for 1 1/2 hours with horse red blood cells (0.5% cells in saline solution) to detect antibody titres against H5N1 viruses. The serum-virus mixture was incubated for 45 minutes with turkey red blood cells (0.5% in saline solution) to detect antibody responses against H1N1-2009. HI antibody titres were scored as the reciprocal of the highest dilution that did not exhibit agglutination of red blood cells.

### Neutralizing Antibody (NAB) Assay

The same RDE-treated samples were also assayed for the presence of virus-neutralizing antibodies. Serum was diluted two-fold down a 96-well round-bottom microtitre plate, starting at an initial dilution of 1/10 in MEM (0.1% BSA, pen/strep). Diluted sera were combined with 100 plaque forming units (pfu) of H5N1-H05 or H5N1-HK97 virus and incubated at 37°C with 5% CO_2_ for 1 hour (total volume of 100 µl). The mixture was transferred onto a subconfluent monolayer of MDCK cells in a 96-well flat bottom microtitre plate and incubated at 37°C with 5% CO_2_ for 5–10 minutes. MEM (with 0.1% BSA and pen/strep) containing 1 µg/ml TPCK-trypsin was then added to each well and the assay was incubated for 48 hours at 37°C with 5% CO_2_. The reciprocal of the highest serum dilution that did not exhibit cytopathic effects (CPE) was recorded as positive for NAB.

Hemagglutination inhibition and neutralizing antibody assays were performed on sera from 8–10 individual mice. All work involving *in vitro* infections was performed at the BSL-4 containment facility at the National Microbiology Laboratory/Public Health Agency of Canada (NML/PHAC).

### Anti-HBsAg Antibodies

Groups of 8 BALB/c mice were vaccinated with 1 µg of a commercially available Hepatitis B (Engerix-B, GlaxoSmithKline) combined with 50 µg of free peptide or without peptide. Serum was collected at 2, 4, 6, and 8 weeks post-immunization and anti-HBsAg antibodies were evaluated. An enzyme-linked immunosorbant assay (ELISA) was performed for each set of serum samples. Serum was diluted two-fold down a 96-well microtitre plate that had been pre-coated with capture antigen. Antibodies were detected using anti-mouse antibodies conjugated to horse-radish peroxidase and visualized using a colorimetric system. ELISA plates were read at optical density of 450 nm (OD450 nm). Serum samples were evaluated separately for each individual mouse and the assay was repeated in triplicate. The data is presented as the arithmetic mean titre along with standard error of the mean.

### Human Toll-like Receptor and NOD-like Receptor Screening

Peptides were sent to Invivogen for screening of pattern recognition receptor activation. Briefly, peptides were added onto HEK 293 cells expressing specific TLR or NOD receptors. After 16–20 hours of incubation, cells were evaluated for TLR or NOD receptor induction via the reporter gene secreted alkaline phophatase (SEAP), which is under the control of transcription factor NF-κB. When the TLR or NOD receptor is induced, signalling through the TLR to NF-κB will allow the expression of SEAP. This can be detected and monitored by reading the OD at 650 nm. Peptides were screened using seven human TLRs (TLR2–5, 7–9) at four concentrations (0.05, 0.5, 5, and 50 µg) and two NOD ligands (NOD1 and NOD2) were tested at two concentrations (5 and 50 µg). Assays were performed in duplicate and readouts were provided by the company.

### Flow Cytometry

Natural killer cells were isolated from mouse spleens using magnetic bead separation (EasySep). Cells were cultured in RPMI 1640 medium in the presence of 100 U/ml or 1000 U/ml of recombinant mouse IL2 (RD systems) overnight with 2.5 µg/ml of 5mer4 peptide or 5mer2 peptide and DMSO as controls. NK cells were then harvested after 24 hours and stained with anti-mouse CD3 conjugated to AlexaFluor 488 (AF488), anti-mouse CD69 conjugated to R-phycoerythrin (PE) and DX5 (CD49b) conjugated to allophycocyanin (APC). Activation of NK cells by peptide was monitored by the percentage of CD3^−^ DX5^+^ CD69^+^ cells. NK isolation and incubation with peptide was repeated in three independent experiments.

### NK Depletion

Depletion of NK cells in BALB/c mice was performed by administration of rabbit anti-mouse asialo antibody (Wako). Each mouse received two 50 µL intraperitoneal injections at days -2 and -1 before vaccination. NK depletion was confirmed one day following vaccination (70–80% depletion). Groups of 8 mice were then administered the H5N1-HA DNA vaccine with or without 50 µg of 5mer4 peptide and challenged with 100LD_50_ of lethal homologous H5N1-H05 virus 28 days following immunization.

### Statistics

All weight and survival data was compiled with GraphPad 5.0 software and analysed for statistical differences using two-tailed unpaired t-tests or one-way analysis of variance (ANOVA). The Mantel-Cox test was used to analyse survival curves. Differences between treatment and control groups was considered significant when p<0.05.

## Supporting Information

Figure S1 **Frequency of 5-mers in the Universal Proteome.** A graphical distribution comparing number of 5-mer peptides and occurrence in the UniProtKB database.
(TIF)

Figure S2 **Western blot probing H5N1-HA expression of each short peptide conjugated to H5-HA** ***in cis*** **.** Each 5-mer, 9-mer, and 13-mer construct that was incorporated into the H5N1-HA gene was transfected into HEK 293T cells using calcium phosphate reagents. Cells were harvested after 48 hours and lysed using radioimmunoprecipitation buffer. Equivalent protein amounts were loaded and expression probed using a polyclonal anti-H5HA mouse primary antibody followed by a goat anti-mouse antibody conjugated to horseradish peroxidase. Protein bands were visualized using ECL reagent (GE Healthcare). M = Marker, Lane 1 = untransfected control, Lane 2 = pCAGα transfected negative control, Lane 3 = pCAGα-HA positive control, Lane 4 = HA-5mer1, Lane 5 = HA-5mer2, Lane 6 = HA-5mer3, Lane 7 = HA-5mer4, Lane 8 = HA-5mer5, Lane 9 = HA-5mer6, Lane 10 = 9mer1, Lane 11 = 9mer3, Lane 12 = 9mer4, Lane 13 = 13mer1, Lane 14 = 13mer3, Lane 15 = 13mer4.
(TIF)

Table S1 **List of 5mers, 9mers, and 13mers evaluated.** All peptides evaluated are organized by sequence, hydrophobicity, and organism in which each sequence is found. †Organisms are organized by Kingdom.
(PDF)
