# Supplementary figures and images for: Pentamers Not Found in the Universal Proteome Can Enhance Antigen Specific Immune Responses and Adjuvant Vaccines
Source: PLoS One. 2012 Aug 24;7(8):e43802. doi: 10.1371/journal.pone.0043802 (PMC3427150; doi:10.1371/journal.pone.0043802)

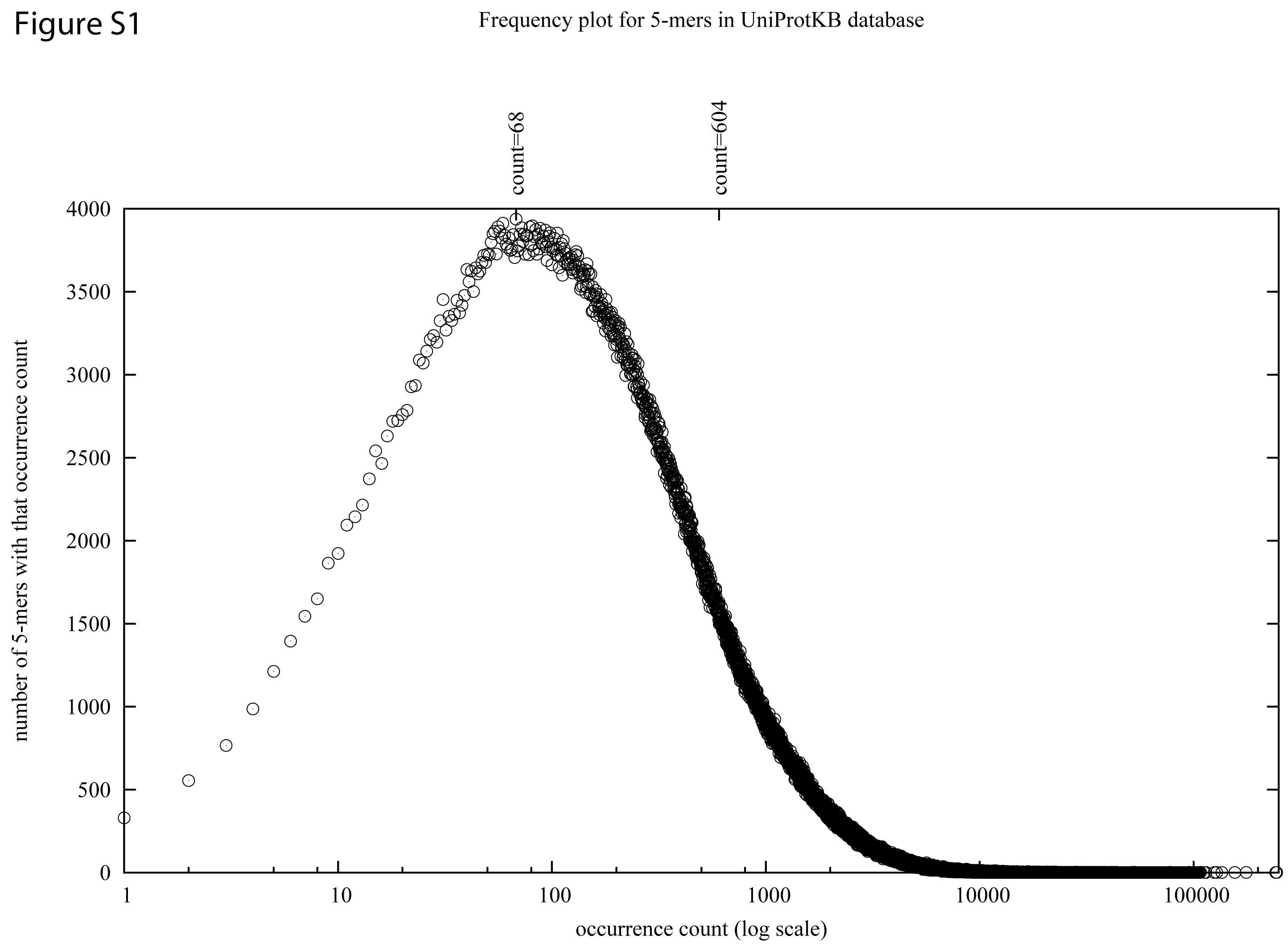

Supplement: Figure S1 — Frequency of 5-mers in the Universal Proteome. A graphical distribution comparing number of 5-mer peptides and occurrence in the UniProtKB database. (TIF) [file pone.0043802.s001.tif]

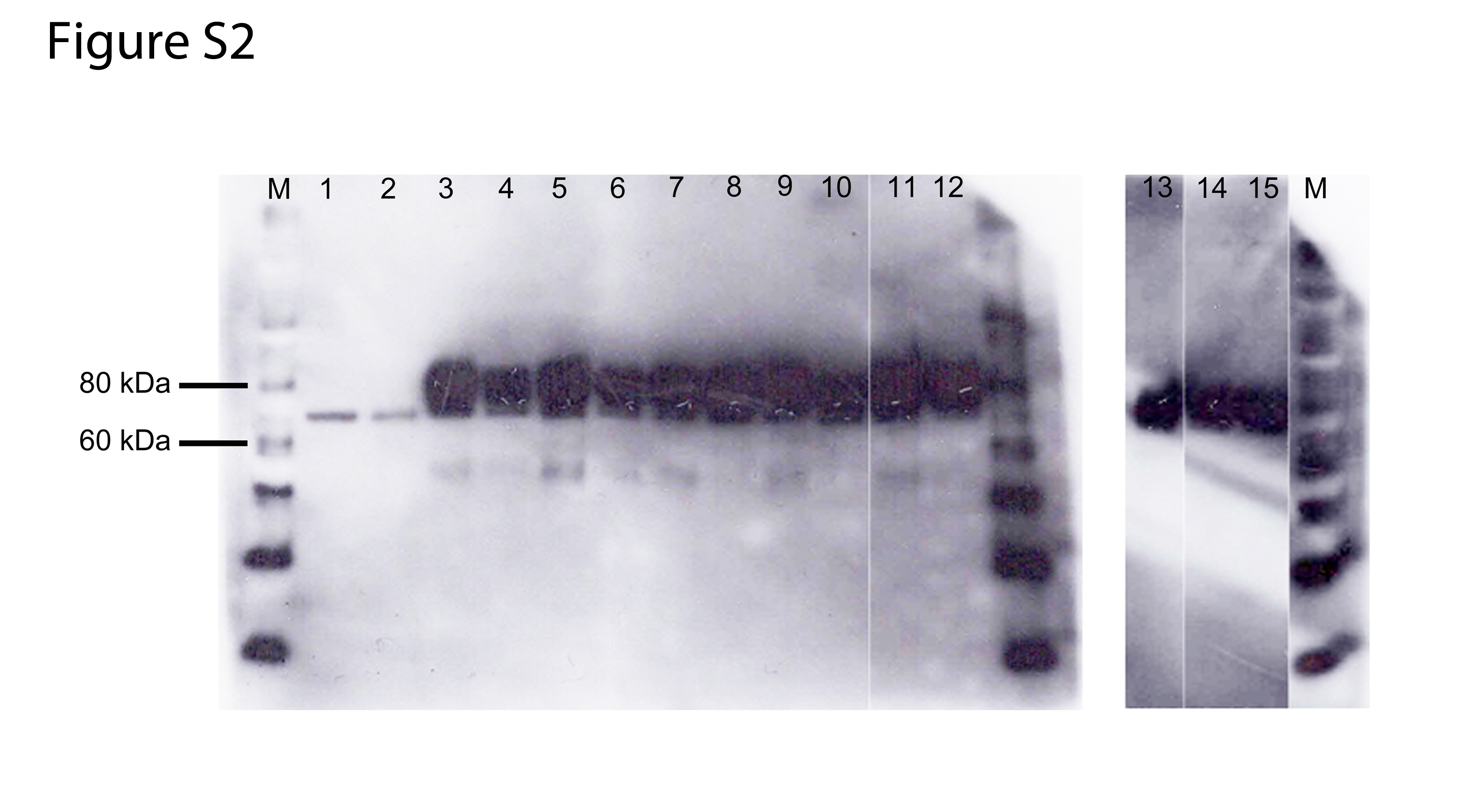

Supplement: Figure S2 — Western blot probing H5N1-HA expression of each short peptide conjugated to H5-HA in cis . Each 5-mer, 9-mer, and 13-mer construct that was incorporated into the H5N1-HA gene was transfected into HEK 293T cells using calcium phosphate reagents. Cells were harvested after 48 hours and lysed using radioimmunoprecipitation buffer. Equivalent protein amounts were loaded and expression probed using a polyclonal anti-H5HA mouse primary antibody followed by a goat anti-mouse antibody conjugated to horseradish peroxidase. Protein bands were visualized using ECL reagent (GE Healthcare). M = Marker, Lane 1 = untransfected control, Lane 2 = pCAGα transfected negative control, Lane 3 = pCAGα-HA positive control, Lane 4 = HA-5mer1, Lane 5 = HA-5mer2, Lane 6 = HA-5mer3, Lane 7 = HA-5mer4, Lane 8 = HA-5mer5, Lane 9 = HA-5mer6, Lane 10 = 9mer1, Lane 11 = 9mer3, Lane 12 = 9mer4, Lane 13 = 13mer1, Lane 14 = 13mer3, Lane 15 = 13mer4. (TIF) [file pone.0043802.s002.tif]
