# Supplementary material for: Pentamers Not Found in the Universal Proteome Can Enhance Antigen Specific Immune Responses and Adjuvant Vaccines
Source: PLoS One. 2012 Aug 24;7(8):e43802. doi: 10.1371/journal.pone.0043802 (PMC3427150; doi:10.1371/journal.pone.0043802)
Supplement: Table S1 — List of 5mers, 9mers, and 13mers evaluated. All peptides evaluated are organized by sequence, hydrophobicity, and organism in which each sequence is found. †Organisms are organized by Kingdom. (PDF) [file pone.0043802.s003.pdf]

Table S1: 5mers, 9mers, and 13mers evaluated

Name	Sequence	Hydrophobicity	Organism†
5mer1	CHKWD	20%	**<u>Eukarya</u>**<br>**Fungi:**<br>*Puccinia graminis* (1-phosphatidylinositol-3-phosphate 5-kinase)<br>*Naumovozyma dairenensis* (hypothetical protein)<br>**Plantae:**<br>*Melampsora larici-populina* (hypothetical protein)<br>*Brachyscome latisquamea* (maturase)<br>*Brachyscome ciliocarpa* (maturase)<br>**Animalia:** *Amphimedon queenslandica* (hypothetical protein)<br>**<u>Bacteria</u>**<br>*Opitutaceae bacterium* (hypothetical protein)<br>*Peptostreptococcus anaerobius* (transcriptional regulatory, LytR family)<br>*Denitrovibrio acetiphilus* (hypothetical protein)<br>**<u>Archaea</u>**<br>*Nitrosopumilus maritimus* (hypothetical protein)<br>*Ramlibacter tataouinensis* (hypothetical protein)<br>*Candidatus Nitrosoarchaeum limnia* (phospho-2-dehydro-3-deoxyheptonate aldolase)
5mer2	WHKCE	20%	**<u>Eukarya</u>**<br>**Fungi:** *Pyrenophora tritici-repentis* (predicted protein)<br>*Metarhizium anisopliae* (hypothetical protein)<br>**Chromalveolata**: *Blastocystis hominis* (unnamed protein product)<br>**Animalia:** *Nematostella vectensis* (predicted protein)<br>*Ornithorhynchus anatinus* (Platypus, hypothetical protein)<br>*Drosophila yakuba* (GE10666)<br>**Plantae:** *Gluconacetobacter diazotrophicus* (hypothetical protein)<br>*Populus trichocarpa* (predicted protein)<br>**Unranked:** *Monosiga brevicollis* (hypothetical protein)<br>**<u>Bacteria</u>**<br>*Gluconacetobacter diazotrophicus* (Superfamily I DNA and RNA helicase and helicase subunits-like protein)<br>*Agrobacterium tumefaciens* (acetyltransferase)<br>*Capnocytophaga* sp (hypothetical protein)<br>*Clostridium hathewayi* (hypothetical protein)
5mer3	CKWRC	20%	**<u>Eukarya</u>**<br>*Sporisorium reilianum* (related to high-affinity nickel transport protein nic1)<br>*Pyrenophora tritici-repentis* (hypothetical protein)<br>Neurospora tetrasperma (hypothetical protein)<br>**Animalia:** *Ciona intestinalis* (predicted similar to Sodium channel protein type 5 subunit alpha)<br>*Brugia malayi* (Ubiquitin carboxyl-terminal hydrolase family protein)<br>*Loa loa* (hypothetical protein)<br>*Caenorhabditis remanei* (hypothetical protein)<br>*Osmerus mordax* (Centaurin-alpha 2)<br>*Sabellaria alveolata* (fructose-bisphosphate aldolase)

			Limnodrilus sp (fructose-bisphosphate aldolase)
5mer3			**Plantae:** *Vitis vinifera* (hypothetical protein)<br>*Selaginella moellendorffii* (hypothetical protein)<br>*Physcomitrella patens subsp. patens* (predicted protein)<br>Volvox carteri f. nagariensis (hypothetical protein)<br>**Bacteria**<br>*Neisseria bacilliformis* (hypothetical protein)
**5mer4**	KWCEC	20%	**Eukarya**<br>**Excavata:** *Leishmania mexicana* (hypothetical protein)<br>**Chromalveolata**: Cryptosporidium muris (hypothetical protein)<br>**Plantae :** *Physcomitrella patens subsp. patens*, *Populus trichocarpa* (conserved hypothetical protein)<br>**Bacteria**<br>*Bathycoccus sp., Paenibacillus curdlanolyticus*, *Clostridium nexile*, *Clostridium ljungdahlii*, *Bacteroides pectinophilus*, *Rhodococcus erythropolis*, *Veillonella* sp., *Ahrensia* sp. (conserved hypothetical protein)<br>*Atopobium rimae* (methyltransferase)
5mer5	DCWMD	40%	**Eukarya**<br>**Chromalveolata**: *Emiliania huxleyi* (ankyrin-1-like protein)<br>**Fungi:** *Candida tenuis* (hypothetical protein)<br>*Ajellomyces sp.* (C6 transcription factor)<br>*Paracoccidioides brasiliensis* (hypothetical protein)<br>*Trichophyton sp.* (C6 transcription factor)<br>*Arthroderma sp* (C6 transcription factor)<br>**Animalia:** *Pediculus humanus corporis* (hypothetical protein)<br>*Daphnia pulex* (hypothetical protein)<br>*Taeniopygia guttata* (hypothetical protein)<br>**Plantae:** *Chlorella variabilis* (hypothetical protein)<br>**Bacteria**<br>*Asticcacaulis biprosthecum* (pectinesterase family protein)<br>*Bacteroides sp.* (Glycoside hydrolase)<br>*Clostridium sp* (hypothetical protein)<br>*Bacillus megaterium* (allantoate amidohydrolase)<br>*Parvibaculum lavamentivorans* (beta-lactamase-like protein)<br>Agrobacterium sp. (competence protein F)<br>*Campylobacter gracilis* (hypothetical protein)<br>*Bordetella petrii* (glutathione S-transferase)<br>**Virus**<br>Duck reovirus GZ/CHN/2007 (S3 protein)<br>Grapevine leafroll-associated virus 7 (heat shock protein 70)
**5mer6**	KYMCW	60%	**Eukarya**<br>*Ciona intestinalis* (hypothetical protein)<br>**Bacteria**<br>*Desulfobacterium* sp. (hypothetical protein)
9mer1	CWKCWCMFE	44%	Not found in any proteome
9mer3	WNWCMHWDC	44%	Not found in any proteome
9mer4	WHWCMMCWD	56%	Not found in any proteome
13mer1	HEHWCMMWHCCMI	46%	Not found in any proteome
13mer3	HMMCHWMCWCDMH	46%	Not found in any proteome
13mer4	CHMMCHWMWCCMD	46%	Not found in any proteome
